# Colony-stimulating factor 1 receptor (CSF1R) inhibitors in cancer therapy

**DOI:** 10.1186/s40425-017-0257-y

**Published:** 2017-07-18

**Authors:** Michael A. Cannarile, Martin Weisser, Wolfgang Jacob, Anna-Maria Jegg, Carola H. Ries, Dominik Rüttinger

**Affiliations:** Roche Pharmaceutical Research and Early Development, Roche Innovation Center Munich, Nonnenwald 2, Penzberg, 82377 Germany

**Keywords:** CSF1, CSF1R, Tumor-associated macrophage, Clinical trial, Cancer therapy, PVNS, Dt-GCT

## Abstract

The tumor-permissive and immunosuppressive characteristics of tumor-associated macrophages (TAM) have fueled interest in therapeutically targeting these cells. In this context, the colony-stimulating factor 1 (CSF1)/colony-stimulating factor 1 receptor (CSF1R) axis has gained the most attention, and various approaches targeting either the ligands or the receptor are currently in clinical development. Emerging data on the tolerability of CSF1/CSF1R-targeting agents suggest a favorable safety profile, making them attractive combination partners for both standard treatment modalities and immunotherapeutic agents. The specificity of these agents and their potent blocking activity has been substantiated by impressive response rates in diffuse-type tenosynovial giant cell tumors, a benign connective tissue disorder driven by CSF1 in an autocrine fashion. In the malignant disease setting, data on the clinical activity of immunotherapy combinations with CSF1/CSF1R-targeting agents are pending. As our knowledge of macrophage biology expands, it becomes apparent that the complex phenotypic and functional properties of macrophages are heavily influenced by a continuum of survival, differentiation, recruitment, and polarization signals within their specific tissue environment. Thus, the role of macrophages in regulating tumorigenesis and the impact of depleting and/or reprogramming TAM as therapeutic approaches for cancer patients may vary greatly depending on organ-specific characteristics of these cells. We review the currently available clinical safety and efficacy data with CSF1/CSF1R-targeting agents and provide a comprehensive overview of ongoing clinical studies. Furthermore, we discuss the local tissue macrophage and tumor-type specificities and their potential impact on CSF1/CSF1R-targeting treatment strategies for the future.

## Background

**Fig. 1:**
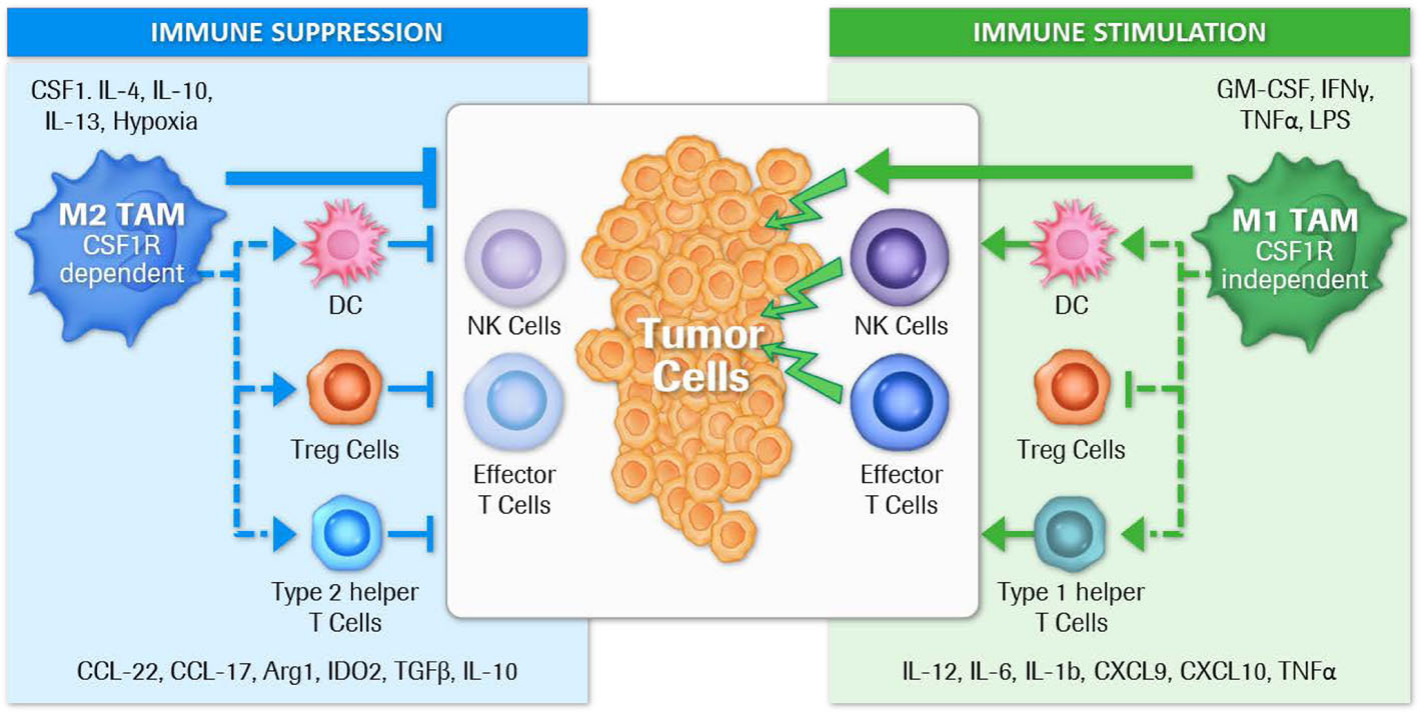
Direct and indirect regulation of immune suppression or stimulation by tumor associated macrophage subtypes. Macrophage polarization within the tumor microenvironment is highly dependent on the local cytokine milieu which originates either from tumor cells, other stromal cells such as immune cells or fibroblasts, as well as macrophages themselves. The M2 TAM phenotype is a consequence of the continuous presence of growth factors such as colony-stimulating factor-1 (CSF1) as well as CD4+ T cell-derived T_h_2 cytokines interleukin (IL)-4, IL-13 and IL-10 (5). Besides the direct tumor growth promoting abilities of M2 TAM (not illustrated here), these macrophages efficiently suppress immune effector functions that are able to contribute to tumor cell elimination (3,4). This silencing of immune effector cells is achieved by producing cytokines and enzymes that may directly suppress effector cells or indirectly via other immune cell types such as intratumoral dendritic cells (DC), T regulatory cells (Treg cells) and Type 2 helper T cells. In contrast, M1 TAM are attributed with tumoricidal functions and are generated in the presence of GM-CSF and pro-inflammatory stimuli like IFNγ, LPS or TNFα (5). Tumoricidal function can either be achieved through direct killing of tumor cells or by producing cytokines/chemokines that are activating/recruiting other immune stimulatory immune cells and inhibiting immune suppressive cells like Treg cells. Eventually a predominant M1 TAM phenotype may result in an anti-tumor immune effector cell activation. Published data suggest that tumor promoting and immune suppressive M2 macrophages/TAM are dependent on CSF1R mediated signals (31) making this receptor an attractive target to eliminate or repolarize these cells

Macrophages are known to be a highly plastic cell type that adapts to the particular stromal environment present in malignant tumors, characterized by tissue necrosis, low oxygen pressure, and high concentrations of lactate and pyruvate [1]. Macrophages have been described as responding to this micromilieu with either a pro-inflammatory or an anti-inflammatory phenotype (also referred to as “fight” versus “fix” macrophages, respectively) [2]. In early stage as well as metastatic cancer, the dominant tumor-associated macrophage (TAM) phenotype is reported to be anti-inflammatory, immune-regulatory, and therefore tumor-promoting (also termed alternatively activated or M2 macrophages) as opposed to pro-inflammatory and tumoricidal (classically activated or M1 macrophages). We and others believe that the continuum of different macrophage phenotypes present within the tumor microenvironment (TME) is difficult to capture solely with the M1/M2 dichotomy. However, for simplicity reasons, we use the term M1 or M2 macrophage/TAM to differentiate two extreme functional phenotypes in this review. M2 macrophages/TAM have been reported to promote tumor growth, angiogenesis, invasion, and metastasis as well as resistance to therapy [3, 4]. In addition, TAM infiltration has been shown to have a negative prognostic relevance in most tumor types [5]. This phenotype is a consequence of the continuous presence of growth factors such as colony-stimulating factor-1 (CSF1; or macrophage colony-stimulating factor [MCSF]) as well as the cluster of differentiation (CD)-4^+^ type 2 helper T-cell-derived (T_h_2) cytokines interleukin (IL)-4, IL-13, and IL-10 in the TME. In contrast, M1 macrophages are ascribed tumoricidal functions and are generated in the presence of granulocyte-macrophage colony-stimulating factor (GM-CSF or CSF2) and pro-inflammatory stimuli such as interferon (IFN)-γ, lipopolysaccharide, or tumor necrosis factor α [6] (Fig. 1).

CSF1 receptor (CSF1R)-mediated signaling is crucial for the differentiation and survival of the mononuclear phagocyte system and macrophages in particular [7]. CSF1R belongs to the type III protein tyrosine kinase receptor family, and binding of CSF1 or the more recently identified ligand, IL-34, induces homodimerization of the receptor and subsequent activation of receptor signaling [8]. As the intratumoral presence of CSF1R^+^ macrophages correlates with poor survival in various tumor types [5, 9], targeting CSF1R signaling in tumor-promoting TAM represents an attractive strategy to eliminate or repolarize these cells.

In addition to TAM, CSF1R expression can be detected on other myeloid cells within the tumor microenvironment such as dendritic cells, neutrophils, and myeloid-derived suppressor cells (MDSCs)

For the latter, Holmgaard and colleagues provided evidence for MDSC reprogramming towards a proinflammatory, tumoricidal phenotype upon treatment with a CSF1R small-molecule inhibitor, PLX3397 [10]. However, a clear interpretation of the role of MDSCs in inflammatory responses remains challenging because of the phenotypic, morphological, and functional heterogeneity of these cells in mice and humans [11]. As our understanding of the influence of CSF1/CSF1R-mediated signaling on human myeloid-derived cells other than macrophages is just emerging, the focus of this review is on TAM and current clinical efforts to specifically target CSF1/CSF1R in cancer therapy. We also highlight the importance of site/organ and tumor-type specificities of TAM, which are now recognized as an important new frontier in cancer immunotherapy. Early clinical data suggest good tolerability of CSF1/CSF1R-targeting compounds; however, available efficacy data are still limited, with the exception of compelling anti-tumor activity observed in diffuse-type tenosynovial giant cell tumors (dt-GCT), a benign connective tissue disorder driven by CSF1 in an autocrine fashion [12]. The individual CSF1R inhibitors and their different drug-targeting properties have recently been reviewed [13]. Only two clinical-stage programs are currently targeting CSF1. No molecules targeting IL-34, the second-known ligand for CSF1R, are in clinical development thus far. We use “CSF1R inhibitor” as a general term for both receptor- and ligand-targeting compounds.

## Clinical activity with CSF1R inhibitor monotherapy

A variety of small molecules and monoclonal antibodies (mAbs) directed at CSF1R or its ligand CSF1 are in clinical development both as monotherapy and in combination with standard treatment modalities such as chemotherapy as well as other cancer-immunotherapy approaches (Tables 1–3).

**Table 1:** CSF1/CSF1R inhibitors as monotherapy in current clinical development

Class	Target	Compound	Clinical Phase	Sponsor	Indication	ClinicalTrials.gov identifier	Status/Results	Reference
Small molecules	CSF1R (and cKIT, Flt3)	Pexidartinib (PLX3397, PLX108-01)	2	The Christie NHS Foundation Trust	KIT-mutated advanced acral and mucosal melanoma	NCT02071940	Ongoing	-
			1/2	Plexxikon	Unresectable or metastatic KIT-mutated melanoma	NCT02975700	Ongoing	-
			2	Plexxikon	Advanced castration-resistant prostate cancer with bone metastasis and high circulating tumor cell counts	NCT01499043	Not yet reported	-
			2	Plexxikon	Recurrent GBM	NCT01349036	ORR: 0% CBR: 7/38 (18%)	[14, 26]
			1/2	NCI	Refractory leukemias and refractory solid tumors, including neurofibromatosis type 1-associated plexiform neurofibromas	NCT02390752	Ongoing	-
			2	Plexxikon	Relapsed or refractory cHL	NCT01217229	ORR: 1/20 (5%)	[15]
			1/2	Plexxikon	Relapsed or refractory FLT3-ITD-positive acute myeloid leukemia	NCT01349049	Ongoing	-
			1	Plexxikon	Advanced, incurable, solid tumors in which the target kinases are linked to disease pathophysiology	NCT01004861	Ongoing	-
	CSF1R (and Trk)	PLX7486	1	Plexxikon	Solid tumors	NCT01804530	Ongoing	-
	CSF1R	ARRY-382	1	Array BioPharma	Solid tumors	NCT01316822	ORR: 0% CBR: 4/26 (15%)	[17]
	CSF1R	JNJ-40346527	1/2	Johnson& Johnson	cHL	NCT01572519	ORR: 1/21 (5%) CBR: 11/21 (52%)	[16]
	CSF1R	BLZ945	1/2	Novartis	Solid tumors	NCT02829723	Ongoing	-
Monoclonal antibodies	CSF1R	Emactuzumab (RG7155)	1	Roche	Solid tumors	NCT01494688	PMR: 5/44 (11%) ORR: 0% CBR: 6/40 (24%)	[18]
	CSF1R	AMG820	1	Amgen	Solid tumors	NCT01444404	ORR: 1/25 (4%) CBR: 6/25 (24%)	[19]
	CSF1R	IMC-CS4 (LY3022855)	1	Eli Lilly	Solid tumors	NCT01346358	Ongoing	-
			1	Eli Lilly	Breast and prostate cancer	NCT02265536	Ongoing	-
	CSF1	MCS110	1/2	Novartis	Prostate cancer	NCT00757757	Terminated	-

*CBR* clinical benefit rate, *cHL* classical Hodgkin lymphoma, *CSF1* colony-stimulating factor 1, *CSF1R* colony-stimulating factor 1 receptor, *GBM* glioblastoma, *NCI* National Cancer Institute, *NHS* National Health Service, *ORR* objective response rate, *PMR* partial metabolic response

Among the class of small molecules, pexidartinib (PLX3397), an oral tyrosine kinase inhibitor of CSF1R, cKIT, mutant fms-like tyrosine kinase 3 (FLT3), and platelet-derived growth factor receptor (PDGFR)-β, is the subject of the broadest clinical development program in monotherapy, with completed or ongoing studies in c-kit-mutated melanoma, prostate cancer, glioblastoma (GBM), classical Hodgkin lymphoma (cHL), neurofibroma, sarcoma, and leukemias. Additional CSF1R-targeting small molecules, including ARRY-382, PLX7486, BLZ945, and JNJ-40346527, are currently being investigated in solid tumors and cHL. mAbs in clinical development include emactuzumab, AMG820, IMC-CS4, cabiralizumab, MCS110, and PD-0360324, with the latter two being the only compounds targeting the ligand CSF1.

A phase 2 study in 38 patients with recurrent GBM treated with pexidartinib did not show significant improvement in 6-month progression-free survival (PFS) compared to historical control data. Of 38 patients, seven (18%) experienced stable disease; no partial or complete responses were observed [14]. An objective response rate (ORR) of 5% was reported with single agent PLX3397 in 20 heavily pre-treated patients with cHL [15]. Comparable efficacy in relapsed or refractory cHL was demonstrated with JNJ-40346527 in a phase 1/2 clinical study. Out of 21 patients enrolled, one showed a complete response (ORR 5%) and 11 (52%) experienced stable disease [16].

Results from a phase 1 study investigating ARRY-382 in advanced solid tumors were recently reported by Bendell et al. Out of 26 patients, four (15%) had stable disease, and no objective responses were observed [17]. A phase 1/2 study with BLZ945 in solid tumors is ongoing.

**Fig. 2:**
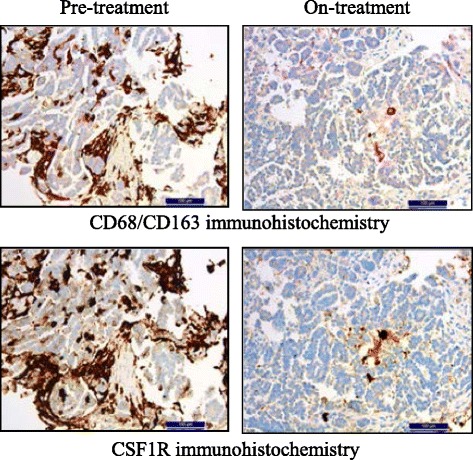
Depletion of tumor-associated macrophages with emactuzumab in cancer patients. Immunohistochemistry of paired tumor biopsies from a representative ovarian cancer patient illustrating co-localization and reduction of CD68^+^CD163^+^ TAM (upper panel) and CSF1R^+^ cells (lower panel) after 4 weeks/two infusions of emactuzumab at the 1000 mg dose level. Permission for re-use granted by I. Klaman [18]

Data from a phase 1 dose-escalation and expansion study investigating emactuzumab showed partial metabolic responses in fluorodeoxyglucose-positron emission tomography in 5/44 (11%) patients and stable disease by Response Evaluation Criteria in Solid Tumors (RECIST) in 6/40 (15%) patients [18]. In addition, the study provided proof of mechanism, demonstrating significant TAM reduction with emactuzumab in paired pre- and on-treatment tumor biopsies (Fig. 2).

Papadopoulos et al. reported that 6/25 patients (24%) treated with AMG820 had a best overall response of stable disease, and one paraganglioma patient (4%) had a partial response, with a 40% reduction in tumor burden [19].

Results from two ongoing single-agent phase 1 studies of IMC-CS4 in solid tumors and breast and prostate cancer are pending. A phase 1/2 study in prostate cancer of the only anti-CSF1 antibody, MSC110, has been terminated; however, several clinical trials are underway with MSC110 in combination with chemotherapy or immune checkpoint inhibitor therapy (Tables 2 and 3).

## Anti-tumor activity of CSF1R inhibitors in diffuse-type tenosynovial giant cell tumor (dt-GCT)

dt-GCT of the soft tissue (alternatively known as pigmented villonodular synovitis [PVNS]) is an orphan disease characterized by overexpression of CSF1 and is usually caused by chromosomal translocations involving chromosome 1p13 where the CSF1 gene is located. CSF1R activation leads to the recruitment of CSF1R-expressing macrophages that constitute a large part of the tumor mass in dt-GCT, thus making this pathway an ideal therapeutic target for compounds interfering with the CSF1/CSF1R-signaling axis. Unresectable dt-GCT is rarely, if ever, a lethal disease but rather a debilitating chronic illness of high unmet medical need, frequently requiring several surgical procedures.

Initial clinical activity was seen in a dt-GCT patient who was treated with the BCR-ABL tyrosine kinase-targeting agent imatinib (Gleevec®) and achieved a complete response [20]. Thereafter, several phase I studies tested CSF1R-targeting compounds in selected dt-GCT patients as a proof-of-concept disease (Table 4). Substantial clinical activity was observed in the study by Cassier et al. (2015), in which 22 of 28 patients (79%) treated with the CSF1R-targeting mAb emactuzumab achieved a partial response, two patients (7%) had a complete response, three patients (11%) had stable disease, and no patient had disease progression [12]. Another study showed unconfirmed partial responses in four of four patients (100%) treated with the CSF1-targeting mAb MCS110 [21]. With the small-molecule inhibitor pexidartinib, 12 of 23 patients (52%) had a partial response, seven (30%) had stable disease, and one (4%) had progressive disease [22]. Responses in these studies were durable (>1 year for pexidartinib and >1.9 years for emactuzumab), and the median PFS had not been reached at the time of publication [12, 22]. A phase 3 study has started in patients with dt-GCT or giant cell tumor of the tendon sheath (GCT-TS) treated with pexidartinib versus placebo (ClinicalTrials.gov identifier NCT02371369). Although exciting clinical activity with CSF1R inhibition has been confirmed in dt-GCT, the safety profile of CSF1R-targeting compounds needs to be considered carefully in this non-life-threatening disease (see the next section for a detailed safety discussion).

**Table 2:** Clinical trials with CSF1/CSF1R inhibitors in combination with anti-tumor therapies (excluding cancer-immunotherapy doublets)

Class	Target	Compound	Combination partner	Clinical phase	Sponsor	Indication	ClinicalTrials.gov identifier	Status/Results	Reference
Small molecules	CSF1R (and cKIT, Flt3)	Pexidartinib (PLX3397, PLX108-01)	Androgen-deprivation therapy plus external radiotherapy	1	Plexxikon/Daiichi Sankyo	Prostate cancer	NCT02472275	Ongoing	-
			Paclitaxel	1	Plexxikon/Daiichi Sankyo	Solid tumors	NCT01525602	ORR: 4/23 (17%) CBR: 14/23 (61%)	[35]
			Eribulin	1/2	Plexxikon/Daiichi Sankyo	Breast cancer	NCT01596751	Ongoing	-
			Temozolomide plus external radiotherapy	1/2	Plexxikon/Daiichi Sankyo	GBM	NCT01790503	Not yet reported	-
			Vemurafenib	1	Plexxikon/Daiichi Sankyo	BRAF-mutated melanoma	NCT01826448	Terminated	-
			PLX9486 (KIT inhibitor)	1/2	Plexxikon/Daiichi Sankyo	GIST	NCT02401815	Ongoing	-
			Sirolimus	1/2	Plexxikon/Daiichi Sankyo	Advanced sarcomas, MPNST	NCT02584647	Ongoing, not yet reported	[73]
Monoclonal antibodies	CSF1R	Emactuzumab (RG7155)	Paclitaxel	1	Roche	Ovarian and breast cancer	NCT01494688	Not yet reported	-
	CSF1R	Emactuzumab (RG7155)	Paclitaxel plus bevacizumab	2	Roche	Ovarian cancer	NCT02923739	Ongoing	-
	CSF1	MCS110	Carboplatin plus gemcitabine	2	Novartis	TNBC	NCT02435680	Ongoing	-
	CSF1	PD-0360324	Cyclophosphamide	2	Pfizer	Recurrent platinum-resistant epithelial ovarian, peritoneal, or fallopian tube cancer	NCT02948101	Ongoing	-

*CBR* clinical benefit rate, *CSF1* colony-stimulating factor 1, *CSF1R* colony-stimulating factor 1 receptor, *dt-GCT* diffuse-type tenosynovial giant cell tumor, *GBM* glioblastoma, *GIST* gastrointestinal stromal tumor, *MPNST* malignant peripheral nerve sheath tumor, *ORR* objective response rate, *TNBC* triple-negative breast cancer

In addition to tumor shrinkage as a measure of efficacy, functional and symptomatic improvement is another important aspect to assessing clinical benefit in patients with dt-GCT. Patient-reported outcome measures could add evidence for superior clinical benefit of CSF1R inhibitors over surgery as the current mainstay of therapy. Test instruments such as the Western Ontario and McMasters Universities Osteoarthritis Index (WOMAC) questionnaire, the Brief Pain Inventory (BPI), the worst pain numeric rating scale (NRS), and the Patient-Reported Outcomes Measurement Information System (PROMIS) physical functioning items were introduced into clinical studies to investigate whether tumor shrinkage correlated with clinical benefit for these patients [12, 23]. Preliminary results from 22 patients treated with pexidartinib showed trends toward improvement in both pain and joint stiffness over time [24]

## Clinical safety and tolerability of CSF1R inhibitors

Preliminary safety results from phase 1 and 2 studies have been reported for CSF1R inhibitor monotherapy in a variety of settings, including healthy subjects and patients with rheumatoid arthritis, cHL, or advanced solid tumors. In some studies no dose-limiting toxicities (DLTs) were reported [12, 18, 25], whereas others have observed DLTs defining a maximum tolerated dose (MTD) [17, 19, 22]. Overall, the adverse event (AE) profile of CSF1R inhibitors has been characterized quite extensively for the different compounds. Frequently reported AEs for both small molecules and mAbs include fatigue, elevated liver enzymes, facial and peripheral edema, asthenia, pruritus, rash, nausea/vomiting, headache, dry skin, increased lacrimation, and decreased appetite [12, 17, 18, 22, 26–30]. Increases in creatine kinase, lactate dehydrogenase, aspartate aminotransferase (AST), and alanine transaminase (ALT) were seen across studies [12, 17, 19, 22, 25–27, 30, 31]. Most studies reported that, despite elevations of these enzymes, patients did not experience clinical signs of liver toxicity, and bilirubin levels remained within the normal range [22, 27, 30, 31]. Short-lived elevations of liver enzymes have also been observed in healthy volunteers [28]. The asymptomatic increases in liver enzymes with CSF1R-targeting treatment are most likely caused by a decrease in physiologic clearance through partial depletion of sessile macrophages of the liver (CSF1R^+^ Kupffer cells) [13, 32]. Therefore, liver enzyme elevations can be considered a class effect of CSF1R-targeting compounds. In general, it seems this is not associated with functional liver impairment or structural damage to hepatocytes.

However, there might be differences between CSF1R inhibition with mAbs and with small molecules. For example, although facial edema is reported for up to 64% of patients treated with the mAb emactuzumab [12], it seems to occur to a lesser extent for the small molecule pexidartinib (seen in 26% of patients [22]), and periorbital edema was not reported in a phase 2 study in 63 rheumatoid arthritis patients treated with the small molecule JNJ-40346527 [31]. Potentially immune-related AEs have been described for mAbs [12], whereas serious liver injuries have not been reported. In contrast, enrolment into a phase 3 study with pexidartinib (NCT02371369) was recently suspended because two of 121 patients experienced non-fatal serious liver toxicity [33]. Whether hepatotoxicity may be triggered by inhibition of other receptor kinases, for example, as suggested by hair color changes with pexidartinib treatment in up to 74% of patients (likely due to inhibition of KIT kinase), remains unclear [22]. Potent KIT inhibitors such as dasatinib and pazopanib also cause ALT and AST elevations in about 50% of tumor patients, and hepatocellular necrosis has been shown in patients treated with pazopanib [34]. Hence, it cannot be ruled out that inhibition of tyrosine kinases other than CSF1R contribute to an aggravation of liver toxicities, particularly with small-molecule inhibitors.

In line with the overall favorable safety profile of CSF1R inhibitors, combination treatment studies have been initiated for both chemotherapies and targeted therapies or immunotherapies. For example, pexidartinib was tested together with paclitaxel in solid tumor patients, and no DLTs were reported [35]. As described in the next section, combinations with programmed cell death protein 1 (PD1) and programmed cell death-ligand 1 (PDL1) inhibitors are ongoing. As with most combination therapies, the promise of increased clinical activity is accompanied by the risk of additive toxicity and therefore requires careful assessment. However, the lack of significant overlapping toxicities of the single-agent AE profiles means these two classes of compounds are promising candidates for successful combination strategies.

## Clinical combinations including CSF1R inhibitors

Rational combination therapies investigating CSF1R inhibition have been investigated in preclinical cancer models (recently reviewed by Ries et al. [13]). Various small-molecule kinase inhibitors and antibodies directed against CSF1 or CSF1R were combined with chemotherapies, irradiation, anti-angiogenic or cancer immunotherapies using immunocompetent and immunodeficient mouse models. Notably, the attack of tumor cells via chemotherapy or irradiation induced an upregulation of tumor-derived CSF1 secretion followed by enhanced TAM infiltration that provided additional growth and survival factors for the tumor. A similar mechanism was described for anti-angiogenic therapy that resulted in enhanced supply of vascular endothelial growth factor (VEGF) by TAM. Hence, the combination of tumor-targeted or anti-angiogenic therapies and CSF1R inhibitors resulted in improved anti-tumoral activity. Another important link between TAM and cytotoxic CD8^+^ T cells was established using combinations with adoptive T-cell therapy or immune checkpoint inhibitors. In this context, TAM-derived suppressive cytokines such as IL-10 or the general T-cell suppressive functions of TAM provided the basis for increased tumor-inhibitory effects of CSF1R inhibitors combined with immunotherapies. On the basis of these results, multiple clinical trials combining various CSF1/CSF1R inhibitors with agents of diverse mechanisms of action were initiated. Examples include combinations with radiation and androgen-deprivation therapy in prostate cancer, radiation therapy and temozolomide in GBM, rapamycin in peripheral nerve sheet tumors, paclitaxel and eribulin in breast cancer, vemurafenib in melanoma, and KIT inhibitors in gastrointestinal stromal tumors (GISTs). Details and references are summarized in Table 2.

**Table 3:** Clinical trials with CSF1/CSF1R inhibitors in combination with cancer immunotherapy agents

Class	Target	Compound/Class	Combination partner	Clinical phase	Sponsor	Indication	ClinicalTrials.gov identifier	Status/Results	Reference
Small molecule	CSF1R (and cKIT, Flt3)	Pexidartinib (PLX3397, PLX108-01)	Pembrolizumab (anti-PD1 mAb)	1/2	Plexxikon/Daiichi Sankyo	Solid tumors, malignant melanoma GIST, NSCLC, ovarian carcinoma, TNBC, SCCHN, UBC, pancreatic cancer, gastric carcinoma, leiomyosarcoma, cholangio carcinoma, CRC (MSS)	NCT02452424	Ongoing	[74]
			Durvalumab (anti-PDL1 mAb)	1	Astra Zeneca	Pancreatic carcinoma, CRC	NCT02777710	Ongoing	-
	CSF1R	ARRY-382	Pembrolizumab (anti-PD1 mAb)	1	Array BioPharma	Solid tumors, melanoma, NSCLC	NCT02880371	Ongoing	-
	CSF1R	BLZ945	PDR001 (anti-PD1 mAb)	1/2	Novartis	Solid tumors	NCT02829723	Ongoing	-
Monoclonal antibody	CSF1R	Emactuzumab (RG7155)	Atezolizumab (anti-PDL1 mAb)	1	Roche	Solid tumors, TNBC, gastric cancer, soft tissue sarcoma, UBC, ovarian cancer, NSCLC, melanoma	NCT02323191	Ongoing	-
			RG7876 (CD40 agonist mAb)	1	Roche	TNBC, gastric cancer, mesothelioma, CRC, melanoma, pancreatic cancer	NCT02760797	Ongoing	-
	CSF1R	AMG820	Pembrolizumab (anti-PD1 mAb)	1	Amgen	Solid tumors	NCT02713529	Ongoing	-
	CSF1R	Cabiralizumab (FPA008)	Nivolumab (anti-PD1 mAb)	1	FivePrime/BMS	Solid tumors, NSCLC, SCCHN, pancreatic cancer, ovarian cancer, RCC, GBM	NCT02526017	Ongoing	[75]
	CSF1R	IMC-CS4 (LY3022855)	Durvalumab (anti-PDL1 mAb) or Tremelimumab (anti-CTLA4 mAb)	1	Eli Lilly	Solid tumors	NCT02718911	Ongoing	[76]
	CSF1	MCS110	PDR001 (anti-PD1 mAb)	1/2	Novartis	Solid tumors, TNBC, pancreatic cancer, melanoma, endometrial cancer	NCT02807844	Ongoing	-
	CSF1	PD-0360324	Avelumab (anti-PDL1 mAb)	1	Pfizer	Solid tumors	NCT02554812	Ongoing	-

*CRC* colorectal cancer, *CSF1* colony-stimulating factor 1, *CSF1R* colony-stimulating factor 1 receptor, *CTLA4* cytotoxic T-lymphocyte-associated protein 4, *GBM* glioblastoma, *GIST* gastrointestinal stromal tumor, *mAb* monoclonal antibody, *MSS* microsatellite stable, *NSCLC* non-small cell lung cancer, *PD1* programmed cell death protein 1, *PDL1* programmed cell death ligand 1, *RCC* renal cell carcinoma, *SCCHN* squamous cell carcinoma of the head and neck, *TNBC* triple-negative breast cancer, *UBC* urothelial bladder carcinoma

Clinical trials assessing the combination of CSF1R inhibitors with immune checkpoint inhibition clearly outnumber other ongoing combination efforts. This may be because immune checkpoint inhibitors have revolutionized therapeutic strategies in oncology, due to the durable clinical benefit experienced by a fraction of patients from enhancing systemic anti-tumor immunity. The cytotoxic T-lymphocyte-associated protein 4 (CTLA4) antagonist ipilimumab was the first checkpoint inhibitor to demonstrate clinical activity that led to a first approval in metastatic melanoma in 2011 [36]. Anti-PD1 and anti-PDL1 mAbs such as nivolumab, pembrolizumab, or atezolizumab have proven superior efficacy over standard-of-care therapy in a variety of indications such as melanoma, non-small-cell lung cancer (NSCLC), bladder cancer, and others [37–42]. Despite the sustained and deep responses observed in some patients, the majority of cancer patients do not respond to these agents. The underlying primary and secondary resistance mechanisms are not well understood; however, evidence is increasing that overcoming the immunosuppressive TME is key to improving the clinical activity of cancer immunotherapy. It is well documented that TAM and other myeloid cells contribute to an immunosuppressive TME. CSFR1 blockade has been shown to reduce T-cell-suppressive TAM infiltrates [13, 18]. Therefore, CSF1R inhibitors represent a promising combination partner for T-cell-enhancing immunotherapies. Based on the widespread use of PD1 and PDL1 inhibitors across tumor entities, a variety of clinical trials combining these agents with CSF1R inhibitors have been initiated. Most of these trials are currently in the dose-finding phase or are evaluating safety and preliminary efficacy in expansion cohorts (Table 3). Clinical results are eagerly awaited, and investigators are aiming to establish superiority of the combination regimen over checkpoint inhibition monotherapy, with the ultimate goal of replacing the current standard of care in various tumor types.

**Table 4:** Clinical trials with CSF1R inhibitors for the treatment of dt-GCT

Class	Target	Compound	Clinical phase	Sponsor	Indication	ClinicalTrials.gov identifier	Status/ Results	Reference
Small molecules	CSF1R (and cKIT, Flt3)	Pexidartinib (PLX3397, PLX108-01)	1	Plexxikon	Solid tumors and extension for MEC, dt-GCT, GIST, ATC, metastatic solid tumors	NCT01004861	Ongoing ORR: 12/23 (52%) CBR: 19/23 (83%)	[22]
			3	Plexxikon/Daiichi Sankyo	dt-GCT or GCT-TS	NCT02371369	Ongoing	-
Monoclonal antibodies	CSF1R	Emactuzumab (RG7155)	1	Roche	Solid tumors and dt-GCT	NCT01494688	ORR: 24/28 (86%) CBR: 27/28 (96%	[12]
	CSF1R	Cabiralizumab (FPA008)	1/2	FivePrime	dt-GCT	NCT02471716	Ongoing	-
	CSF1	MCS110	2	Novartis	dt-GCT or GCT-TS	NCT01643850	Ongoing ORR: 4/4 (100%)	[21]

*ATC* anaplastic thyroid cancer, *CBR* clinical benefit rate, *CSF1* colony-stimulating factor 1, *CSF1R* colony-stimulating factor 1 receptor, *dt-GCT* diffuse-type tenosynovial giant cell tumor, *GCT-TS* giant cell tumor of the tendon sheath, *GIST* gastrointestinal stromal tumor, *MEC* mucoepidermoid carcinoma of the lung, *ORR* objective response rate

Apart from combining CSF1R inhibitors with PD1/PDL1 or CTLA4 antagonists, alternative strategies to further enhance anti-tumor efficacy of the host immune system are being investigated. Repolarization of the TME is being pursued by combining the CD40 agonist RO7009789 with the anti-CSF1R antibody emactuzumab in a phase 1 clinical trial (NCT02760797). Preclinically, Mok and coworkers reported that depletion of alternatively activated macrophages with pexidartinib improved the efficacy of adoptive cell transfer in a melanoma mouse model [43]. However, this combination has not been tested clinically. Other approaches include, for example, CSF1R inhibition within a triple combination with chemotherapy and anti-angiogenic treatment in platinum-resistant ovarian cancer (NCT02923739).

## Local tissue macrophage and disease specificity impacting CSF1R-directed treatment strategies

CSF1R-targeting agents exhibit a rather benign safety profile; however, to date, only modest clinical activity as monotherapy has been reported outside of CSF1-driven dt-GCT. Currently, important patient data sets for solid and hematological malignancies, especially those from ongoing combination trials, are still pending. The complex phenotypic and functional properties of macrophages are heavily influenced by a continuum of survival, differentiation, recruitment, and polarization signals within their specific tissue environment. Therefore, a key question remaining to be answered in clinical studies is whether these agents provide benefit to all cancer patients by depleting CSF1R^+^ TAM, or whether certain patients and/or tumor types are more likely to respond to CSF1R inhibition. Evidence is increasing that the individual underlying tumor histology as well as organ site-specific features of CSF1R^+^ cells need to be considered.

In the healthy lung, for instance, resident alveolar macrophages that develop from fetal monocytes are mainly regulated by the presence of local GM-CSF [44, 45]. Downstream of GM-CSF signaling, lung-specific transcription factor peroxisome proliferator-activated receptor (PPAR)-γ and nuclear repressor Bach2 are responsible for surfactant clearance in macrophages that are participating in host defense [46–48]. This supports the hypothesis that alveolar macrophages in healthy steady state are predominantly of the immunostimulatory M1 macrophage subtype. These cells produce T_h_1 cytokines and promote T-cell stimulation while expressing low levels of CSF1R and proving largely resistant to CSF1R inhibitors [13]. Two recent publications report high levels of CSF1R and low human leukocyte antigen-antigen D-related (HLA-DR) as well as increased levels of macrophage markers CD68 and CD163 and decreased levels of inducible nitric oxide synthase (iNOS) on myeloid cells derived from tumors of lung cancer patients [10, 49]. Thus, tumor cells may induce a reprogramming of steady-state alveolar macrophages from the M1, CSF1R^low^ phenotype towards the M2, CSF1R^high^ phenotype, making this tumor type a good candidate for CSF1R-targeting therapies. However, the literature documents contradictory prognostic relevance of TAM in NSCLC [9]. This may in part be due to the different detection methods and markers used, but it is more likely that the contradictory reports indicate differences in the local TAM phenotypes in lung cancer subtypes. Depending on the predominant TAM phenotype, CSF1R inhibition may either a) reprogram an M2 TAM-dominated, immunosuppressive TME through depletion of CSF1R-dependent TAM or b) boost an ongoing anti-tumor response by increasing the M1/M2 TAM ratio in an M1 TAM-dominated TME. For both strategies, the choice of an appropriate combination partner will be key to reprogramming a tumor-promoting TME or boosting a pre-existing anti-tumor immune response. Recently, in vitro-differentiated CD206-expressing human macrophages were shown to be rescued from emactuzumab-induced depletion in the presence of IL-4 [50], demonstrating the importance of the local cytokine micromilieu. As CD206 expression is high on alveolar macrophages, an elevated concentration of IL-4 in lung cancer patients may result in resistance to CSF1R inhibitors. Patient data on local IL-4 concentrations and the effect of CSF1R-targeting agents are not available yet.

The physiological steady state of intestinal macrophages in colon tissue is quite different from those in the lung. Intestinal macrophages also originate from monocytes [51] but exhibit a reduced inflammatory activation state that allows the healthy co-existence with commensal bacteria achieved by locally produced autocrine IL-10 as well as regulatory T-cell-derived IL-10 production [52]. This M2 macrophage phenotype is able to protect colon cancer cells from tumor necrosis factor-related apoptosis-inducing ligand (TRAIL)-induced cell death [53] and is dependent on CSF1R-related signaling, which was demonstrated for different species (i.e., mice, monkeys) and in human colorectal cancer (CRC) patients, where intestinal macrophages were reduced significantly with CSF1R-targeting therapy [13, 18, 54, 55]. This predominant immunosuppressed environment, together with the observed CSF1R dependency of intestinal macrophages, supports using CSF1R inhibition in CRC patients. However, again, available data on the prognostic relevance of macrophages in CRC patients are contradictory [56]. In contrast to stromal macrophages, peritumoral TAM showed an anti-tumor M1 macrophage phenotype in CRC [57]. The pro- or anti-tumor effect of TAM may therefore also depend on their localization within the TME. However, the degree of CSF1R-signaling dependency of macrophages at these different locations is still unclear. In addition to the location within the TME, the genetic stability of tumor cells may influence the general immune status of the TME. In a CRC subset with high microsatellite instability (MSI-high), TAM infiltration correlated with higher immunity compared with microsatellite stable (MSS) tumors [58, 59]. Therefore, the MSI-high subset of patients may represent a promising target population in which to include CSF1R inhibition to boost pre-existing tumor immunity. Despite the lower number of TAM (and immune infiltrates in general) in MSS CRC tumors, CSF1R-targeted therapies may still also be beneficial in this subset of CRC patients. In MSS CRC patients, a higher expression of genes involved with epithelial to mesenchymal transition (EMT) has been reported, an event that is associated with the invasiveness and spreading of tumor cells [60]. In addition to their immunoregulatory capacities, M2 macrophages can be key contributors to the priming of the pre-metastatic niche [61] by, for example, inducing cytokine-mediated EMT [57, 62] and matrix remodeling [63]. Whether CSF1R blockade in MSS CRC patients is able to control the invasiveness and metastasis remains to be investigated.

In contrast to monocyte-derived alveolar and intestinal macrophages, microglia of the central nervous system (CNS) are resident yolk sac-derived macrophages. They are scavengers for non-functional synapses [52, 64, 65], and CSF1R-mediated signaling is required both during early CNS development [66] and for survival in adults [67]. Inhibition of CSF1R signaling via small molecules BLZ9445 [68] or PLX3397 [69] leads to profound depletion of microglia in the CNS without overt behavioral abnormalities or reduced performance in cognitive function in mice. Although of different origin than, for example, intestinal macrophages, the observed function of microglia seem to be similar to those of the M2 macrophage subtype described for intestinal macrophages in the healthy gut. In contrast to the contradictory reports for lung and CRC, the presence of TAM in human gliomas seems to be exclusively associated with tumor growth, grade, and poor prognosis [70, 71]. Targeting of microglia using BLZ9445 resulted in improved survival and regression of tumors in a mouse proneural GBM model. In this tumor model, CSF1R blockade did not result in depletion of microglia as in healthy control mice. Here, the local TME under anti-CSF1R treatment was dominated by tumor cell-derived GM-CSF and IFN-γ. Interestingly, this local cytokine milieu did not result in unresponsiveness of TAM to anti-CSF1R monotherapy treatment but rather in reprogramming of microglia from M2 into M1 TAM. A phase 2 clinical study assessing pexidartinib in GBM patients is currently ongoing (NCT01790503). In the aforementioned GBM in vivo model, long-term anti-CSF1R treatment led to acquired resistance driven by elevated macrophage-derived insulin-like growth factor 1 (IGF-1) and high IGF-1 receptor (IGF-1R) levels on tumor cells, resulting in enhanced glioma cell survival and invasion [72]. Whether high IL-4 levels together with CD206 expression on TAM may also play a role in this acquired resistance to CSF1R inhibition in GBM is currently unknown. Potential translational approaches to prevent or resolve resistance to CSF1R inhibition may consist of treatment schedules other than continuous administration of CSF1R inhibitors. Alternative schedules could, for example, pursue an initial anti-CSF1R-mediated debulking of M2 TAM followed by other treatment modalities to maintain or induce tumor immunity.

With several CSF1R-targeting therapies currently under evaluation in the clinic, we are still only beginning to understand which covariates impact on macrophage phenotypes and the respective role of CSF1R-mediated signaling in cancer. The aforementioned examples illustrate that the origin and the presence of a predominant macrophage phenotype in healthy tissue cannot alone predict pro- or anti-tumor effects of TAM during tumorigenesis. Furthermore, it is difficult to identify tumor types that might or might not benefit from CSF1R-targeted therapies without taking into account further sub-classification of tumors and their respective impact on the local TME. Both primary and disseminated tumor cells may induce a profound functional reprogramming of resident tissue macrophages by changing the local cytokine milieu. Only once the impact of organ-specific CSF1R blockade is better understood will a more precise selection of anti-CSF1R-containing treatment regimens and prediction of clinical benefit for patients be possible.

## Conclusions

CSF1R inhibitors represent an exciting new class of immune-modulatory drugs. Scientific understanding of macrophage and CSF1R biology is evolving rapidly, and more data from clinical trials investigating CSF1R-directed therapies will become available shortly. Whereas clinical tolerability seems to have been established for this group of agents, it is increasingly clear that the organ site and tumor-type specifics of TAM will need to be considered for the selection of both the right patients population and the appropriate combination partner to achieve a meaningful clinical benefit for cancer patients.

## Abbreviations

AE: Adverse event
ALT: Alanine aminotransferase
AST: Aspartate aminotransferase
ATC: Anaplastic thyroid cancer
BPI: Brief Pain Inventory
CBR: Clinical benefit rate
cHL: Classical Hodgkin lymphoma
CNS: Central nervous system
CRC: Colorectal cancer
CSF1: Colony-stimulating factor 1
CSF1R: Colony-stimulating factor 1 receptor
CTLA4: Cytotoxic T-lymphocyte-associated protein 4
DC: Dendritic cells
DLT: Dose-limiting toxicity
dt-GCT: Diffuse-type tenosynovial giant cell tumors
EMT: Epithelial to mesenchymal transition
FLT3: fms-like tyrosine kinase 3
GBM: Glioblastoma
GCT-TS: Giant cell tumor of the tendon sheath
GIST: Gastrointestinal stromal tumor
GM-CSF: Granulocyte-macrophage colony-stimulating factor
HLA-DR: Human leukocyte antigen-antigen D related
IDO: Indolamin-2,3-Dioxygenase
IFNγ: Interferon γ
IGF-1: Insulin-like growth factor 1
IHC: Immunohistochemistry
IL: Interleukin
iNOS: Inducible nitric oxide synthase
mAb: Monoclonal antibody
MCSF: Macrophage colony-stimulating factor
MDSC: Myeloid derived suppressor cells
MEC: Mucoepidermoid carcinoma of the lung
MPNST: Malignant peripheral nerve sheath tumor
MSI: Microsatellite instability
MSS: Microsatellite stable
MTD: Maximum tolerated dose
NRS: Numeric rating scale
NSCLC: Non-small cell lung cancer
ORR: Objective response rate
PD1: Programmed cell death protein 1
PDGFR: Platelet-derived growth factor receptor
PDL1: Programmed cell death-ligand 1
PFS: Progression-free survival
PPAR-γ: Peroxisome proliferator-activated receptor-γ
PROMIS: Patient-Reported Outcomes Measurement Information System
PVNS: Pigmented villonodular synovitis
RCC: Renal cell carcinoma
SCCHN: Squamous cell carcinoma of the head and neck
TAM: Tumor-associated macrophages
Th cells: T helper cells
TME: Tumor microenvironment
TNBC: Triple-negative breast cancer
TNFα: Tumor necrosis factor α
TRAIL: Tumor necrosis factor related apoptosis inducing ligand
UBC: Urothelial bladder carcinoma
VEGF: Vascular Endothelial Growth Factor
WOMAC: Western Ontario and McMasters Universities Osteoarthritis Index
